# Focal Crescentic Glomerulonephritis Following COVID-19 Viral Vector Vaccination

**DOI:** 10.7759/cureus.35305

**Published:** 2023-02-22

**Authors:** Jacqueline M Wisnik-Rainville, Robert Czyzewski, Cynthia Demola, Konrad Czyzewski

**Affiliations:** 1 Department of Internal Medicine, Poznan University of Medical Sciences, Poznan, POL; 2 Department of Internal Medicine, Division of Nephrology, Linden Medical Group, Linden, USA; 3 Department of General Nephrology, John F. Kennedy University Medical Center at Hackensack Meridian Health (HMF), Edison, USA

**Keywords:** covid-19 vaccination, kidney transplant, end stage kidney disease, glomerulonephritis, glomerular disease, acute kidney injury, vaccination, sars-cov2, covid-19

## Abstract

Vaccination is a vital tool aimed at curbing the COVID-19 pandemic, and the FDA has authorized several vaccines for emergency use to combat COVID-19. Our patient presented with acute kidney injury two weeks after receiving the first dose of the COVID-19 Janssen (Johnson & Johnson) vaccine. Renal biopsy confirmed focal crescentic glomerulonephritis. The patient has been unable to achieve remission after diagnosis and is now a candidate for a kidney transplant. In conclusion, this case report provides insight into the possible relationship between glomerular disease following COVID-19 Janssen (Johnson & Johnson) vaccination. Based on this presented case, new-onset or relapse of glomerular diseases presenting post-COVID-19 vaccination should be observed as a possible adverse event to large-scale COVID-19 vaccination.

## Introduction

Immediate and large-scale COVID-19 vaccination has been one of the essential methods used to contain the COVID-19 pandemic. The US FDA issued emergency use authorization for three vaccines to combat the COVID-19 pandemic: Pfizer-BioNTech, Moderna, and Janssen/Johnson & Johnson. To date, >13 billion doses of various COVID-19 vaccines have been administered globally in response to the pandemic. Every day, more and more people are vaccinated, with current data approaching approximately 80.6% of the US population having received at least one dose of the authorized vaccines, with 68.9% of the US population completing the primary series of vaccines [1]. Vaccination against COVID-19 has been an extremely effective tool in preventing COVID-19 infection. Available data reports that the vaccines may cause: mild local side effects, some systemic events, and a very low incidence of severe adverse reactions [2,3,4]. Side effects that occur most frequently include fever, malaise, and injection site pain, and the majority diminish with analgesics and antipyretics. More severe side effects of the COVID-19 vaccines include anaphylaxis and thrombosis. As data is collected from the worldwide vaccination campaign, new reports reveal that there may be a connection between COVID-19 vaccination and the pathogenesis of new-onset or relapsed glomerular diseases [5,6,7]. This case report summarizes a rare case of a previously healthy patient presenting with focal crescentic glomerulonephritis and kidney failure following the administration of the vectored COVID-19 vaccine, Ad26.COV.2.

## Case presentation

A 24-year-old Hispanic-American male with a past medical history of hypertension and sleep apnea presented to the ED for evaluation of chest pain, elevated blood pressure (BP), severe headache, dizziness, and vision changes. He described the persistent headache as increasing in intensity for the past week. When visiting an urgent care center a few days prior to the presentation, the patient was also noted to have elevated BP. He complained of nausea but denied vomiting, diarrhea, or dysgeusia. Vital signs were elevated upon admission: BP 170/105, pulse 91 bpm, respiratory rate 18, temperature 97.7F, SpO2 97%, and BMI 29.9. Physical examination was insignificant for any upper or lower extremity pitting edema, petechiae, jaundice, or rash. The patient was not on any medication prior to his hospitalization, but his past medical history was significant for hypertension, sleep apnea, and uncomplicated bariatric gastric sleeve surgery in 2019. He denied any history of renal disease, including renal stones or even UTIs. A review of his creatinine in 2019 was normal at 1.0. He denied current smoking or alcohol use. The patient was two weeks post receiving his first Ad26.COV.2 (Janssen/Johnson & Johnson) vaccine for COVID-19. Accelerated hypertension and acute renal failure were diagnosed. The patient was admitted to the hospital for BP control and monitoring and referred to the nephrology team. The principal problem remained as accelerated hypertension. Laboratory analysis was remarkable for serum creatinine of 6.5 (0.50-1.6 mg/dL), thrombocytopenia of 55 10*3/uL (120-400 10*3/uL), and estimated glomerular filtration rate (eGFR) of 11 mL/min/1.73m*2 (non-African-American >60 mL/min/1.73m*2) (Table 1). The blood slide review was abnormal, demonstrating decreased platelets, moderate 2+ anisocytosis, moderate 2+ microcytes, and moderate 2+ polychrocytes. Urinalysis was positive for glycosuria, hematuria, RBCs (dysmorphic), and heavy proteinuria >=500. Microscopic urinalysis displayed RBC 11 /HPF (ref. range <3/high power field [HPF]) and trace mucus (ref. range negative/low power field [LPF]).

**Table 1:** Laboratory and serologic parameters on admission. eGFR: Estimated glomerular filtration rate; BUN: Blood urea nitrogen.

Laboratory/Serology parameters	Values	Reference range
WBC	10.5 10*6/uL	4.0-11.0 10*3/uL
RBC	3.37 10*6/uL	4.00-5.80 10*6/uL
Hemoglobin	10.3 g/dL	12.6-17.0 g/dL
Hematocrit	29.2%	42.0-52.0%
Platelet Count	55 10*3/uL	120-400 10*3/uL
Erythrocyte Sedimentation Rate (ESR)	17 mm/h	0-15 mm/h
Sodium	136 mmol/L	133-145 mmol/L
Potassium	3.2 mmol/L	3.3-5.1 mmol/L
Glucose	106 mg/dL	74-106 mg/dL
BUN	55 mg/dL	6-20 mg/dL
Albumin	4.1 g/dL	3.2-4.8 g/dL
Bilirubin, Total	1.9 mg/dL	0.1-1.2 mg/dL
Creatinine	6.5	0.5-1.60 mg/dL
eGFR, African-American	14 mL/min/1.73m*2	> 60 mL/min/1.73m*2
eGFR, Non-African-American	11 mL/min/1.73m*2	> 60 mL/min/1.73m*2

Anti-nuclear antibody, anti-neutrophilic cytoplasmic antibody, anti-glomerular basement membrane (GBM) antibody, and antimyeloperoxidase (MPO) antibody were all negative. Both complement C3 and C4 levels were within the normal range. HIV antigen/antibody, Hepatitis B surface antigen, and Hepatitis C antibody serologies were all negative. Real-time reverse transcriptase-PCR assay for SARS-CoV-2, Influenza A, Influenza B, and Respiratory Syncytial Virus (RSV) were all negative. CT scans of the head, neck, chest, abdomen, and pelvis were ordered to rule out aneurysm and dissection. The CT was done without contrast because laboratory studies demonstrated the patient was in acute renal failure. CT studies demonstrated normal non-contrast head CT, bilateral non-obstructing renal calculi, and gastric sleeve surgery. Chest radiography demonstrated no pulmonary involvement. Following the Nephrology consult, the differential diagnosis was wide, but it was suspected that the acute renal failure was possibly due to acute glomerulonephritis. Other differential diagnoses included: thrombotic thrombocytic purpura (TTP), hypertensive retinopathy, and hemolytic uremic syndrome (HUS). In the meantime, the patient was referred to Interventional Radiology (IR) for placement of a non-tunneled catheter and was ordered to receive hemodialysis (HD) the day after admission. The Nephrology team was hesitant to immediately obtain a renal biopsy due to the higher risk of bleeding complications after a renal biopsy associated with thrombocytopenia. Therefore, the patient's low platelets had to be addressed prior to the biopsy. Hematology/Oncology was consulted, and the patient was scheduled to receive plasmapheresis. After plasmapheresis, the PT count was 127 (normal range: 130-400 10*3/uL) and subsequently continued to rise. With the approval of the Hematology/Oncology team, a renal biopsy was ordered, and all necessary documents were prepared. On the day of the procedure, the patient's platelet count was 234 10*3/uL (120-400 10*3/uL). A biopsy was performed 10 days after admission. A renal biopsy was subsequently performed on the left kidney and showed tubular atrophy, interstitial fibrosis, and moderate interstitial inflammation. It also demonstrated pauci-immune type, focal crescentic glomerulonephritis, with mild activity and mild-to-moderate chronicity. Immunofluorescence revealed that IgG, IgA, IgM, C3, and fibrinogen were all negative, which provided evidence against the glomerular disease of the immune-complex type, and the findings of focal crescent formation indicated small vessel vasculitis. The patient was diagnosed with acute nephritic syndrome and focal crescentic glomerulonephritis, possibly triggered by Ad26.COV.2 (Janssen/Johnson & Johnson) vaccine administration. He was unable to achieve remission following hemodialysis treatment, ACE inhibitor administration, high-dose corticosteroids, cyclophosphamide, and rituximab therapy x 2 doses. The patient did not have an improvement in renal function in the months following their hospital admission; therefore, they were referred to the transplant team and are scheduled to receive a living-related renal transplant (LRRT).

## Discussion

Ad26.COV2.S is a replication-incompetent recombinant adenovirus type 26 (Ad26) vector expressing the SARS-CoV-2 spike protein in a stabilized conformation [8]. The spike protein triggers a response from the body's immune system to start producing antibodies to COVID-19. Non-replicating vector vaccines are unable to make new viral particles; therefore, they only produce the vaccine antigen. The Janssen/Johnson & Johnson vaccine and the Oxford/AstraZeneca vaccines both use viral vectors rather than the mRNA technology used for the Pfizer and Moderna vaccines. Ad26.COV2.S is administered as a single intramuscular injection. The FDA reported that a single-dose injection is 66% effective in preventing moderate-to-severe COVID-19 and 100% effective in preventing COVID-19-related hospitalization and death [8]. Reported adverse effects include thromboembolic events, tinnitus, urticaria, headache, fatigue, myalgia, and vaccination site hypersensitivity. There was a brief pause, suggested by the CDC and FDA, for using the Janssen/Johnson & Johnson vaccine after reports of thrombosis with thrombocytopenia syndrome (TTS) were reported. Although the updated benefit-risk assessment accounted for Guillain-Barre Syndrome (GBS) and TTS, the Advisory Committee on Immunization Practices (ACIP) concluded that the benefits of vaccination with the Janssen COVID-19 vaccine outweighed the risks [9]. However, the ACIP recommended preferential use of mRNA COVID-19 vaccines over the Janssen COVID-19 vaccine; and has recommended for the Janssen vaccine be considered in some situations, including for individuals for whom the mRNA COVID-19 vaccines are contraindicated [9]. Crescentic glomerulonephritis is characterized by rapid loss of renal function with the presence of extensive glomerular crescents (greater than 50%) as the principal histologic finding [10]. Clinically, crescentic glomerulonephritis is characterized by nephritic syndrome, rapidly progressing to end-stage renal disease (ESRD). The etiology and initial pathogenic factors are different in the various types of crescentic glomerulonephritis. Although etiologies vary, the final result consists of damage to the glomerular basement membrane (GBM) [11]. Despite immunosuppressive therapy, individuals carry an increased risk for ESRD and death if, at presentation, they present with renal failure [10]. Another important factor at presentation is serum creatinine, which is directly correlated to the patient's prognosis [11]. Anti-neutrophil cytoplasmic antibodies (ANCA) are proteins made by the immune system which mistakenly target neutrophils and are a type of autoantibody. There is a strong association between glomerular disease and ANCA. The two main types of ANCA are p-ANCA, which targets myeloperoxidase (MPO), and c-ANCA, which targets proteinase 3 (PR3). Although ANCA-negative vasculitis mechanism and pathogenesis remain unclear, some studies have proposed a pathogenic role for ANCAs in developing small vessel vasculitis [12]. Neutrophil infiltration in pathologic lesions of ANCA-negative disease occurs independently of circulating ANCAs and may potentially involve other unidentified autoantibodies or T-cell-dependent mechanisms [12]. ANCA-negative pauci-immune crescentic glomerulonephritis has been identified, suggesting that ANCA is not essential for generating glomerular lesions [12]. Supportive and specific therapies are the focus of treatment and are necessary to resolve crescentic glomerulonephritis. Prompt diagnosis and early treatment are the most vital and critical for patients with crescentic glomerulonephritis. With early intervention, 85%-90% of patients are able to achieve remission within two to six months, but overall, 75% achieve complete remission [10]. Progression of crescentic glomerulonephritis to ESRD can be stopped and lead to complete remission with rapid diagnosis and proper treatment. Supportive therapy consists of managing present infections, volume moderation and monitoring (specifically if dialysis is necessary), and smoking cessation [10]. Specific crescentic glomerulonephritis treatment therapies consist of induction and maintenance of remission. The current mainstay treatment for remission induction remains cyclophosphamide and steroids; however, particular patients qualify for other therapies. Rituximab therapy and plasma exchange are examples of other therapies that can be utilized [10]. Immunosuppressive therapy, such as azathioprine or methotrexate, is encouraged as relapses can be expected [10].

During kidney biopsy of nephropathies, the chronicity is determined by assessing several factors, such as the extent of glomerulosclerosis, tubular atrophy, interstitial fibrosis, and vascular stenosis [13]. Therefore, an alarming sign is when a patient's histological changes demonstrate "mild-to-moderate chronicity" approximately 3-4 weeks post-vaccination. Chronic changes generally appear weeks or months after the initiating event or develop slowly without recognizable acute injury [14]. The level of chronicity, along with the decompensating kidney function, shows the impact the initiating event of vaccination may have had on the body. Vaccines have been the foundation of disease eradication and decreased morbidity and mortality. Although rare, reports show various glomerular diseases and acute kidney injuries following immunization with vaccines other than COVID-19, including influenza, pneumococcal, and hepatitis B vaccines [15]. The correlation between glomerular disease onset and COVID-19 vaccination brings to light the risk of glomerular disease after influenza vaccination. The association is so evident that one literature review noted 65 patients across 45 published reports who developed vasculitis following influenza vaccination [16]. A connection between the pathogenesis of vaccine-associated glomerular disease in either influenza or COVID-19 vaccines has not yet been established. Glomerulonephritis cases have been reported and associated with COVID-19 vaccination and COVID-19 infection. Glomerular diseases reported post-COVID-19 infection include podocytopathy, collapsing glomerulopathy, anti-GBM disease, and ANCA-associated vasculitis [17]. Glomerulonephritis associated with COVID-19 infection pathophysiology is complex. Suspected pathophysiology between the two includes direct cytotoxicity in podocytes and immune dysregulation [15]. It has also been hypothesized that the immune and bodily response to the COVID-19 vaccine mimics the body's response to natural COVID-19 infection, therefore causing susceptible patients to develop glomerulonephritis [17].

## Conclusions

Due to the ever-growing worldwide vaccination campaign, more cases of COVID-19 vaccine-associated glomerular disease (CVAGD) will likely be reported; however, a large amount will remain unreported. Regarding the case presented in this report, a potential pathogenic association is suggested due to the condition taking place within days of vaccination and the onset or flare of glomerular disease; however, the coincidental circumstance cannot be excluded. More research and investigation are imperative to find whether the glomerular disease can be secondary to COVID-19 vaccination.
